# Treatment of Ebola Virus Infection with Antibodies from Reconvalescent Donors

**DOI:** 10.3201/eid2103.141838

**Published:** 2015-03

**Authors:** Thomas R. Kreil

**Affiliations:** Global Pathogen Safety, Baxter BioScience, Vienna, Austria

**Keywords:** reconvalescent plasma, Ebola virus, virus inactivation, virus reduction, viruses, transfusion, outbreak, hemorrhagic disease, whole blood, plasma, solvent/detergent treatment

## Abstract

Clinical evidence suggests that antibodies from reconvalescent donors (persons who have recovered from infection) may be effective in the treatment of Ebola virus infection. Administration of this treatment to Ebola virus–infected patients while preventing the transmission of other pathogenic viruses may be best accomplished by use of virus-inactivated reconvalescent plasma.

The largest outbreak of Ebola virus infection to date included 15,935 reported cases and 5,689 deaths as of November 26, 2014 (World Health Organization Ebola Response Roadmap Situation Report Update, http://apps.who.int/iris/bitstream/10665/144498/1/roadmapsitrep_26Nov2014_eng.pdf). Nearly all infections have been reported in West Africa. The outbreak has reminded public health systems of the minimal medical options available for the treatment of persons affected by this disease. Beyond the management of symptoms, no vaccine or proven causal treatment is available, and interventions that are in development remain at early stages.

Supported by scarce yet positive clinical evidence (*1*) and some recent animal model data (*2*), the use of whole blood or plasma transfusions from reconvalescent donors (persons who have recovered from Ebola infection) that contain antibodies to the Ebola virus has received substantial (also media) attention as a treatment alternative. However, several aspects associated with this approach need consideration to potentially enable treatment at a scale reasonably commensurate to the ongoing outbreak and at a level of safety with respect to the possible transmission of viruses that is consistent with currently accepted standards. The primary choice among options would be between use of whole blood or plasma only.

The use of whole blood transfusions is probably the least desirable choice. For this option, a donor would only be able to donate approximately once per quarter; thus, the number of treatment courses that could be collected from any donor would be fairly limited. In addition, the required matching of blood type (ABO) and antigen (Rh negative/positive) in a whole blood unit for transfusion would add a layer of complexity. Whole blood also cannot be treated by any of the currently approved virus-inactivation methods (reviewed in [*3*]), which would leave virus testing as the only option available to prevent the transmission of infectious agents that the donor may carry, particularly HIV. In resource-rich countries, the implementation of serologic testing for HIV, starting in the mid-1980s, greatly reduced the risk for transmission by blood transfusion (*4*), but rare cases still occur despite use of the most sensitive nucleic acid tests (*5*). This aspect is of particular importance because HIV prevalence in adults is ≈1% in 3 of the affected countries, Liberia, Sierra Leone, and Guinea (http://www.unicef.org/infobycountry). 

On a larger scale, the limitations of testing have been highlighted by transmission of West Nile virus (WNV) through blood transfusions in the United States even after implementation of sophisticated nucleic acid testing schemes for the blood supply (*6*). By contrast, the demonstrated WNV inactivation capacity embedded into the manufacturing processes of plasma derivatives (*7*) has effectively prevented WNV transmission, although plasma for fractionation collected and used in the same geographic region is not tested for WNV. 

Many challenges are associated with establishing and operating a virus-testing laboratory in an environment that lacks the equipment infrastructure or trained personnel. Within these circumstances, it is difficult to ensure that predonation test results for “HIV, HBV, HCV, syphilis, and other locally transmitted infections, as applicable” would be generated within 48 hours, or otherwise repeated at donation, as recommended by interim guidance from WHO (http://apps.who.int/iris/bitstream/10665/135591/1/WHO_HIS_SDS_2014.8_eng.pdf). In addition, the economic aspects of such a testing endeavor would appear challenging.

Transfusion of plasma alone would alleviate a number of the concerns inherent in the use of whole blood. Donor-to-recipient matching complexity would be reduced because only blood type compatibility needs to be established for plasma transfusion. In addition, if plasma were collected by plasmapheresis, a donor could, depending on health status, donate up to twice each week or up to 50 times each year, and up to several hundred milliliters of plasma could be collected per donation. Health care infrastructure and cold-storage capability necessary for effective inventory management are now being deployed to the areas affected by the Ebola outbreak, and addressing the logistics around the installation of an automated plasmapheresis capacity, including providing the required training and supplies, has also received support (*8*). Further, the volumes of antibody-containing material that could be collected by this approach are an order of magnitude higher than the volumes available through whole blood collection, which would enable multiple treatments of patients if neutralizing antibody titers, reported to be highly variable in survivors (*9*), were found to be insufficient to stop virus replication after a single transfusion. 

Another possibility is that, if antibody testing could be implemented, screening the general population in affected areas might prove beneficial to identify persons who have seroconverted in response to asymptomatic infection (*10*). These persons would have uncompromised health status and thus could be even more effective plasma donors, although the level of protection afforded by their Ebola virus antibody spectra would have to be verified through collaboration with specialized laboratories.

After collection, plasma from any donor source could be virus inactivated by an approved method, such as S59 + UVA Intercept or riboflavin + UV Mirasol treatment (reviewed in [*3*]) or by a solvent/detergent (SD) treatment (*11*). These methods would enhance the virus safety margin of plasma units for transfusion by several orders of magnitude. The unique robustness of SD in inactivating all the lipid-enveloped viruses tested (*12*) would seem to make this method the preferred choice for removing concerns about transmission of HIV, HBV, HCV or even the Ebola virus itself. The Intercept and Mirasol technologies have shown a somewhat more limited virus inactivation capacity for certain lipid-enveloped viruses (*3*). 

As has been argued, “Capacity building for the collection and testing of sufficient convalescent blood or plasma from recovered Ebola patients is crucial” (*13*). Whereas some testing can safely be replaced by the more broadly effective inactivation approach described, establishing the infrastructure for, for example, sterile pooling and SD treatment of plasma may still present a challenge. As an alternative, individual plasma units could be SD treated in a commercially available, integral disposable processing bag system (*14*), a system that was developed for use in resource-limited blood bank settings.

To provide for a treatment without any matching requirements that would also make higher virus antibody titers available, laboratories could perform fractionation of reconvalescent plasma into hyperimmune intravenous immunoglobulin preparations. The feasibility of this approach has recently been confirmed at commercial scale during the 2009 influenza A(H1N1) pandemic (*15*). The US-based donor population for this preparation was, however, fully qualified and consistent with the stringent standards required for current plasma fractionation, a situation entirely different from the biosafety challenges associated with bringing reconvalescent plasma from the current Ebola-endemic regions into fractionation facilities licensed according to good manufacturing practices. In addition, available manufacturing capacities for the production of plasma derivatives are used already to support the treatment of persons who have hemophilia or immune deficiencies.

For these reasons, the interim WHO guidance on use of blood and plasma for treatment of Ebola virus infection would benefit from inclusion of a chapter on virus inactivation for plasma. As an effective and readily deployable technical approach, SD-inactivated plasma transfusions might even be proposed as the standard of care.

In conclusion, although the continued development of long-term scalable solutions such as a vaccine for Ebola remains critical, existing technology and protocols could help fill the gap. Establishment of a supply of virus-inactivated reconvalescent plasma for treatment of persons infected with this virus may be the most feasible treatment option.
